# Association between Vitamin C Deficiency and Mortality in Patients with Septic Shock

**DOI:** 10.3390/biomedicines10092090

**Published:** 2022-08-26

**Authors:** Jong Eun Park, Tae Gun Shin, Daun Jeong, Gun Tak Lee, Seung Mok Ryoo, Won Young Kim, You Hwan Jo, Gil Joon Suh, Sung Yeon Hwang

**Affiliations:** 1 Department of Emergency Medicine, Samsung Medical Center, School of Medicine, Sungkyunkwan University, Seoul 06351, Korea; 2 Department of Emergency Medicine, College of Medicine, Kangwon National University, Chuncheon 24289, Korea; 3 Department of Emergency Medicine, Asan Medical Center, College of Medicine, University of Ulsan, Seoul 05505, Korea; 4 Department of Emergency Medicine, Seoul National University Bundang Hospital, Seongnam 13620, Korea; 5 Department of Emergency Medicine, College of Medicine, Seoul National University, Seoul 03080, Korea

**Keywords:** sepsis, septic shock, ascorbic acid, mortality

## Abstract

The prognostic value of low vitamin C levels has not been well investigated in patients with septic shock. We aimed to evaluate the association of vitamin C deficiency with mortality in patients with septic shock. We conducted a retrospective analysis of 165 patients with septic shock from a prospective multicenter trial and institutional sepsis registry between April 2018 and January 2020. The primary outcome was 28-day mortality. The patients were categorized into vitamin C deficiency and normal groups based on a vitamin C cutoff level of 11.4 mmol/L. Multivariable Cox regression analysis was performed to examine the association between vitamin C levels and 28-day mortality. A total of 165 patients was included for analysis and 77 (46.7%) had vitamin C deficiency. There was no significant difference in the 28-day mortality rate between the vitamin C deficiency group and the normal group (23.4% (*n* = 18/77) vs. 13.6% (*n* = 12/88), *p* = 0.083). Multivariable Cox proportional hazard analysis showed vitamin C deficiency to be associated with increased risk of 28-day mortality (adjusted hazard ratio, 2.65, 95% confidence interval (CI), 1.08–6.45; *p* = 0.032). Initial vitamin C deficiency was associated with a higher risk of 28-day mortality in patients with septic shock after adjusting for intravenous administration of vitamin C and thiamine, baseline characteristics, laboratory findings, and severity of illness.

## 1. Introduction

Sepsis and septic shock are potentially life-threatening organ failures caused by a dysregulated host response to infection [1] and are a significant global health burden, affecting millions of people and ranking as a leading cause of death worldwide [2,3,4]. Despite recent advances in sepsis care and ongoing research, in-hospital mortality rates remain unacceptably high, ranging from 10 to 30% in sepsis and up to 52.1% in the presence of septic shock [1,2,3,4,5,6,7]. Several biomarkers have been proposed in clinical and experimental studies to aid in early detection, improved monitoring and management, and risk stratification of sepsis and septic shock [8,9,10,11,12].

The pathophysiology of sepsis and septic shock is highly complex, with many aspects still poorly understood. However, one of the main mechanisms is disruption of redox homeostasis and transition to a pro-oxidant state characterized by excessive synthesis of reactive oxygen and nitrogen species (ROS/RNS) [13,14]. The oxidants activate transcription factors that drive the expression of potent inflammatory cytokines and chemokines, resulting in mitochondrial, endothelial, and microvascular dysfunction and, ultimately, multiple organ failure.

Vitamin C has pleiotropic functions on several biological processes involved in the pathophysiology of sepsis [15,16,17,18]: (1) vitamin C can protect cells and tissues from oxidative damage, as it directly scavenges free radicals and ROS/RNS, inhibits the formation of ROS/RNS, and recycles other antioxidants such as α-tocopherol and tetrahydrobiopterin [15]; (2) vitamin C is an essential cofactor for the biosynthesis of endogenous norepinephrine, dopamine, and vasopressin and enhances adrenergic receptor activity [17]; (3) vitamin C enhances chemotaxis, boosts neutrophil phagocytosis and oxidative killing, promotes lymphocyte proliferation, and reduces inflammation and ROS via attenuation of Nuclear factor-κB activation [18,19]; (4) vitamin C protects against endothelial dysfunction by preventing the activation of nicotinamide adenine dinucleotide phosphate-oxidase, lowering the expression of inducible nitric oxide synthase, and increasing the bioavailability of nitric oxide [20]. It can also prevent an increase in endothelial permeability by limiting protein phosphatase 2A activation and maintaining the production of endothelial nitric oxide via endothelial nitric oxide synthase; and (5) vitamin C reduces capillary plugging and microcirculatory flow impairment by reducing the expression of intracellular adhesion molecules [21]. Given the various vital functions of vitamin C in the sepsis pathophysiology, vitamin C deficiency may adversely affect the prognosis of sepsis patients.

Previous studies have reported that vitamin C deficiency was prevalent and was associated with the severity of organ failure and mortality in critically ill patients [22,23,24]. However, the prognostic value of low vitamin C levels has not been well investigated in patients with septic shock. Although vitamin C deficiency is common in critically ill septic patients, the most recent studies found no significant association between low vitamin C level and outcomes, including 28-day mortality and development of shock [25,26]. In the present study, we aimed to determine the prognostic value of vitamin C deficiency in patients with septic shock diagnosed in the emergency department (ED).

## 2. Materials and Methods

### 2.1. Study Design and Population

We conducted a retrospective analysis of data from the ascorbic acid and thiamine effect in septic shock (ATESS) trial and institutional sepsis registry of Samsung Medical Center, a university-affiliated, tertiary referral hospital in Seoul, South Korea. The study protocol and primary analysis of the ATESS trial have been published [27,28]. In brief, this trial was a multicenter, double-blind, randomized, controlled trial conducted in four academic EDs to examine the effects of intravenous vitamin C and thiamine treatment on patients with septic shock. Another dataset was the institutional sepsis registry, which was collected prospectively with the goal of improving the quality of sepsis management. We retrieved the data from this registry between December 2018 and January 2020 with the same inclusion and exclusion criteria and definitions of the ATESS trial to guarantee comparability. Adult patients (19–89 years of age) who presented to an ED and were diagnosed with septic shock during their ED stay were included in this dataset. Patients who met the following criteria were excluded: invasive treatment limitations (e.g., a signed do-not-resuscitate order); an underlying terminal-stage disease; at least 1 g/day of vitamin C or intravenous thiamine prior to enrollment; history of cardiac arrest prior to enrollment; fulfilling the inclusion criteria more than 24 h after ED arrival; declined to participate in the trial (directly or by legal proxy).

During the study period, thiamine was administered in combination with vitamin C. In the ATESS trial cohort, vitamin C and thiamine administration was determined by randomization, whereas in the institutional sepsis registry cohort, vitamin C and thiamine administration was at the discretion of the treating physician. Other than these vitamins, all patients in the study were treated in accordance with the most recent sepsis and septic shock treatment guidelines (e.g., Surviving Sepsis Campaign: International Guidelines for the Management of Sepsis and Septic Shock), which included timely antibiotic therapy, early source control, hemodynamic optimization with fluid and vasopressor administration, and steroid therapy for refractory shock.

The Institutional Review Board of Samsung Medical Center approved this study (IRB No. 2022-04-127-001) and waived the need for informed consent because the study included anonymized data and was retrospective in nature.

### 2.2. Measurement of Vitamin C

After obtaining informed consent, whole blood was collected from each participant through venipuncture and was placed in a serum-separating tube (SST) containing a clot activator (Vacutainer SST II Tube 8.5 mL, #368972; BD). Following blood collection, blood samples in the SSTs were left for 10 min to coagulate. Blood was centrifuged at 3500 revolutions per minute for 5 min at 25 °C. Serum was separated and kept in aliquots at −70 °C until analysis. Vitamin C concentration in aliquots from SSTs was evaluated using high-performance liquid chromatography coupled with an ultraviolet detector chemical analyzer (PerkinElmer, Waltham, MA, USA).

### 2.3. Data Collection and Definitions

We collected the following data for this study: demographic characteristics including age and sex; past medical history; suspected infection focus (respiratory, intra-abdominal, urinary, others, and undetermined); vital signs (systolic blood pressure (BP), diastolic BP, heart rate, and body temperature) at triage; laboratory tests including the Sequential Organ Failure Assessment (SOFA) score; treatment measures including steroid, vasopressor, renal replacement therapy (RRT); and mechanical ventilation (MV) and clinical outcomes including intensive care unit (ICU) admission, shock reversal, hospital length of stay (LOS), and mortality.

Septic shock was determined based on the Sepsis-3 criteria as sepsis with persistent hypotension requiring vasopressors to maintain a mean arterial pressure of 65 mmHg and a serum lactate level >2 mmol/L despite adequate fluid challenge [1]. Sepsis was defined as an acute increase in total SOFA score ≥2 attributable to infection. If the baseline SOFA score was unknown, it was presumed to be 0. Vitamin C deficiency was defined as blood vitamin C level <11.4 mmol/L. Based on vitamin C level measured upon presentation to the ED, patients were divided into the vitamin C deficiency group (those with vitamin C deficiency) and the normal group (those without vitamin C deficiency).

### 2.4. Outcome Measures

The primary outcome was 28-day mortality. The secondary outcomes were 7-day mortality, in-hospital mortality, change in SOFA score, use of MV or RRT, presence of shock reversal, duration of shock reversal time, ICU-free days, vasopressor-free days, MV-free days, and hospital LOS. The first 14 days from the day of enrolment were chosen as the time frame for the calculation of the free-day-related variables. If the patient died during the first 14 days, this metric was recorded as zero days after death.

### 2.5. Statistical Analysis

Continuous data were presented as the median with interquartile range (IQR), and categorical data were presented as a count with percentage. The Wilcoxon rank-sum and chi-square tests were used as appropriate. The Kaplan–Meier curves for each group were plotted and compared using the log-rank test. To examine the hazards associated with 28-day mortality, we performed univariable and multivariable Cox proportional hazards regression. Patients were followed for 28 days to determine the time to the first event or death. Adjusting confounders were selected a priori with clinical relevancy including baseline characteristics, laboratory findings, and severity of illness, while the presence of vitamin C deficiency was forced in the model. The following variables were included in the final model: vitamin C deficiency, intravenous administration of vitamin C and thiamine, age, chronic liver disease, hematologic malignancy, initial serum lactate, C-reactive protein (CRP), serum albumin, and initial SOFA score. We assessed the proportional hazard assumption using the Schoenfeld residuals plot and Schoenfeld test and found no severe violation. The results are presented as hazard ratio (HR) with 95% confidence interval (CI). Calculations were performed with STATA 17 (Stata, College Station, TX, USA) and R version 4.0 (R Foundation for Statistical Computing, Vienna, Austria). Statistical significance was set at *p* < 0.05.

## 3. Results

### 3.1. Baseline Characteristics

**Figure 1:**
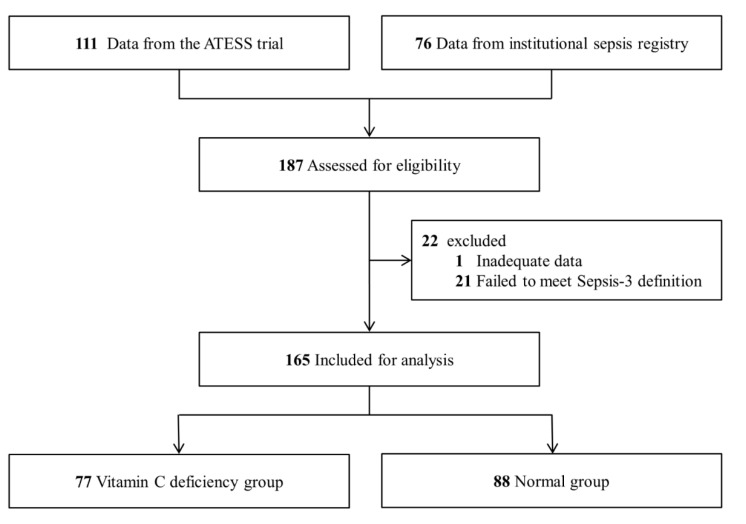
Study flowchart.

A total of 187 patients was screened, and 165 patients were included in the final analysis after excluding those who did not meet the inclusion criteria and whose samples were missing (Figure 1). The baseline characteristics of the study population are shown in Table 1. Among the 165 patients, 77 (46.7%) had vitamin C deficiency. The median age was 70 years (61–76 years), and 62.4% (*n* = 103/165) were male. Chronic liver disease (15.6% vs. 3.4%; *p* = 0.015) and hematologic malignancy (22.1% vs. 8.0%; *p* = 0.019) were more frequent in the vitamin C deficiency group than in the normal group. Initial lactate and follow-up lactate after fluid resuscitation were not significantly different between the groups. Initial SOFA score was significantly higher in the vitamin C deficiency group than in the normal group (8.0 (IQR, 7.0–10.0) vs. 7.0 (IQR, 5.5–10.0); *p* = 0.004). The median vitamin C was 5.1 mmol/L (IQR, 3.0–7.7 mmol/L) in the vitamin C deficiency group and 23.4 mmol/L (IQR, 15.9–41.1 mmol/L) in the normal group (*p* < 0.001).

### 3.2. Outcomes

**Figure 2:**
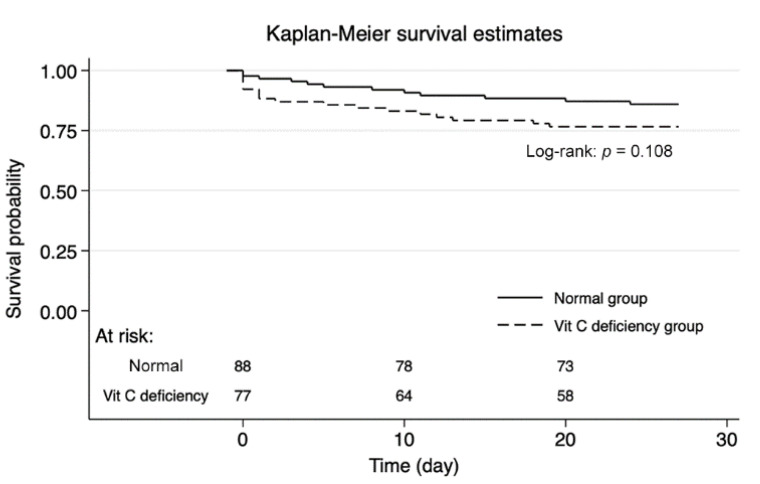
Kaplan–Meier analysis for 28-day mortality.

The primary and secondary outcomes are shown in Table 2. Overall, 28-day mortality was 18.1%. There was no significant difference in the 28-day mortality rate between the vitamin C deficiency group and the normal group (23.4% (*n* = 18/77) vs. 13.6% (*n* = 12/88), *p* = 0.083). Secondary outcomes were not significantly different between the groups. The Kaplan–Meier survival plot for 28-day mortality showed no significant differences in survival patterns between patients with and without vitamin C deficiency (*p* = 0.108; Figure 2).

The results from univariable and multivariable Cox regression analyses for 28-day mortality are shown in Table 3. Vitamin C deficiency was not a significant risk factor for 28-day mortality in univariable analysis (HR 1.80; 95% CI, 0.87–3.73; *p* = 0.115). After adjusting for IV administration of vitamin C and thiamine, baseline characteristics, laboratory findings, and severity of illness, patients with vitamin C deficiency had a significantly higher risk of 28-day mortality than subjects in the normal group (adjusted HR, 2.65; 95% CI, 1.08–6.45; *p* = 0.032).

## 4. Discussion

This study evaluated the association of vitamin C levels and 28-day mortality using a prospectively collected dataset of patients with septic shock who presented to the ED. Results showed that approximately half of the patients had vitamin C deficiency, which was significantly associated with an increased risk of 28-day mortality.

Low plasma vitamin C level is prevalent in critically ill patients, particularly those with sepsis and septic shock. According to Iglesias et al., 50% of sepsis and septic shock patients admitted to an ICU (*n* = 137) had hypovitaminosis C, and 14% had vitamin C deficiency [23]. In a study of 44 critically ill patients (24 with septic shock) admitted to the ICU, Carr et al. showed that 68% had hypovitaminosis C (<23 μmol/L) and 32% had vitamin C deficiency (<11 μmol/L) [22]. Patients with septic shock had significantly lower levels of vitamin C than subjects without septic shock (15.3 ± 7.9 vs. 20.8 ± 8.9 μmol/L; *p* = 0.03). In addition, more patients with septic shock had hypovitaminosis C than non-septic patients (88% vs. 50%), and more patients with septic shock had vitamin C deficiency than non-septic patients (38% vs. 25%).

There are several possible explanations for the low plasma vitamin C level. Massive production of cytokines, one of the hallmarks of sepsis, may impair cellular control of vitamin C absorption, leading to depletion of intracellular vitamin C. Subramanian et al. demonstrated that lipopolysaccharide decreases intestinal vitamin C absorption by downregulating expression of sodium-dependent vitamin C transporter-1 and -2 (SVCT-1 and SVCT-2) [29]. Seno et al. also reported that the activity of SVCT-2, which is functionally expressed in human endothelial cells, is negatively regulated by inflammatory cytokines, such as tumor necrosis factor-α and interleukin-1β, which play a significant role in the pathophysiology of sepsis [30]. In addition, low plasma vitamin C levels can be caused by an increase in metabolic consumption as well as a decrease in the recycling of dehydroascorbic acid due to massive amounts of oxidative stress [31]. By providing electrons, vitamin C protects cells and tissues from oxidative damage. When there is an excessive amount of oxidative stress, there is a rapid depletion of vitamin C. Thus, the level of oxidative stress may have a significant impact on vitamin C level. Given that the CRP or early SOFA score was significantly higher in the vitamin C deficiency group in our study, the initial low vitamin C level appears to reflect the higher oxidative stress and severe inflammation and organ injury than in the normal group.

Several studies have been conducted in different clinical settings to examine the association between low plasma vitamin C concentration and patient outcomes, and controversial results were obtained. In a study of 16 patients who were admitted to a surgical ICU, Borrelli et al. showed that vitamin C level was significantly decreased in patients who were more likely to suffer from multiple organ failures across all ICU days [32]. However, in a recent study by Prasad et al. of 235 patients with severe sepsis, initial vitamin C level was not associated with outcomes including 28-day mortality and development of shock [25]. In a subgroup of 203 patients without pre-existing liver disease, low plasma vitamin C level was strongly associated with increased 28-day mortality, although this association was no longer significant after 60 days or one year. In contrast to the study by Prasad et al., our study showed a significant association of vitamin C deficiency with an increased risk of 28-day mortality. Due to the numerous key roles of vitamin C in pathophysiology and the possible causes of the high prevalence of low plasma vitamin C level in patients with sepsis and septic shock, the initial vitamin C concentration is likely associated with prognosis in patients with septic shock. In addition, we included only patients with septic shock based on the Sepsis-3 definition, resulting in a high disease severity that might explain why our results differed from those of Prasad et al. [25].

As previously stated, vitamin C is an essential nutrient with pleiotropic effects on the multiple biological processes involved in the pathophysiology of sepsis [15]. This pathophysiologic rationale supported the use of vitamin C in patients with septic shock in early studies. Although several researchers have observed a positive effect of vitamin C in combination with thiamine and/or steroids on the outcomes including reduced SOFA score (HYVCTTSSS trial) [33], shorter shock duration (ORANGES trial) [23], and reduced mortality (CITRIS-ALI trial) [34], in other studies such as the VITAMINS trial [35], ACTS trial [36], and ATESS trial [27], no improvement in clinical outcomes was observed. In contrast, a recently published large-scale randomized controlled trial (LOVIT trial) of adults with sepsis receiving vasopressor treatment in the ICU found that the composite of death, continued vasopressor requirement, mechanical ventilation, or renal-replacement therapy on day 28 was significantly higher in those who received intravenous vitamin C than in those who received placebo [37]. Considering the conflicting findings of recent clinical trials, vitamin C deficiency is one of the complex pathophysiologic phenomena in sepsis, but it may not be a critical target of sepsis management, despite the association between vitamin C deficiency and mortality in patients with septic shock indicated by the present results.

The present study had several limitations. First, although prospectively collected data were used, retrospective analyses are associated with inherent limitations. Second, the sample size was small. Consequently, the findings require validation in a larger cohort with a prospective assessment. Third, the matching of the two datasets might not have been precise. Fourth, although vitamin C can be used to predict the prognosis of sepsis patients, its practical applicability in clinical practice might be limited. Rapidly obtaining a result using the most generally utilized detection method is currently difficult; vitamin C testing results are normally available within three business days after submission to the laboratory. Fifth, the conclusion was based on a rather short follow-up period, although 28-day mortality is a commonly used primary measure of sepsis. Examination of other outcomes, such as long-term survival, might yield results that differ from those of 28-day mortality.

## 5. Conclusions

Initial vitamin C deficiency was significantly associated with increased risk of 28-day mortality in patients with septic shock who presented to the ED.

## Figures and Tables

**Table 1:** Baseline characteristics of patients.

	Overall (*n* = 165)	Vitamin C Deficiency (*n* = 77)	Normal (*n* = 88)	*p*-Value
Age, years	70 (61–76)	71 (64–76)	69 (57–73.5)	0.117
Sex, male	103 (62.4)	48 (62.3)	55 (62.5)	0.999
Comorbidities				
Hypertension	70 (42.4)	34 (44.2)	36 (40.9)	0.792
Diabetes	54 (32.7)	24 (31.2)	30 (34.1)	0.816
Cardiac disease	24 (14.5)	9 (11.7)	15 (17.0)	0.452
Chronic lung disease	12 (7.3)	9 (11.7)	3 (3.4)	0.081
Chronic renal disease	10 (6.0)	7 (9.1)	3 (3.4)	0.578
Chronic liver disease	15 (9.1)	12 (15.6)	3 (3.4)	0.015
Hematologic malignancy	24 (14.5)	17 (22.1)	7 (8.0)	0.019
Metastatic solid cancer	59 (35.8)	22 (28.6)	37 (42.0)	0.101
Cerebrovascular disease	15 (9.1)	5 (6.5)	10 (11.4)	0.416
Suspected infection focus				0.383
Respiratory infection	48 (29.1)	22 (28.6)	26 (29.5)	
Intra-abdominal infection	68 (41.2)	35 (45.5)	33 (37.5)	
Urinary tract infection	34 (20.6)	16 (20.8)	18 (20.5)	
Others or unknown	15 (9.1)	4 (5.2)	11 (12.5)	
Laboratory tests				
Lactate (mmol/L)				
On presentation	4.1 (2.8–5.7)	4.1 (3.0–5.4)	3.9 (2.6–5.8)	0.919
After fluid challenge	3.8 (2.6–5.5)	3.7 (2.5–5.3)	3.9 (2.6–5.7)	0.370
WBC (103/μL)	8.00 (1.99–16.1)	6.45 (2.05–16.20)	8.75 (1.78–15.92)	0.485
Hemoglobin (g/dL)	10.4 (9.2–12.6)	10.1 (8.7–12.1)	11.1 (9.3–13.1)	0.059
Platelet count (103/μL)	118 (56–191)	112 (40–190)	124 (68–192)	0.280
Creatinine (mg/dL)	1.4 (1.03–2.12)	1.6 (1.1–2.5)	1.2 (1.0–1.8)	0.007
Albumin (g/dL)	3.0 (2.5–3.4)	2.9 (2.4–3.3)	3.1 (2.5–3.5)	0.216
Albumin level (mg/dL)				0.283
≥3.5	39 (23.6)	14 (18.2)	25 (28.4)	
≥2.5 and <3.5	86 (52.1)	42 (54.5)	44 (50.0)	
<2.5	40 (24.2)	21 (27.3)	19 (21.6)	
Total bilirubin (mg/dL)	0.9 (0.6–2.2)	1.1 (0.6–2.3)	0.8 (0.5–2.0)	0.159
Procalcitonin (mmol/L)	12.6 (2.5–37.4)	10.0 (2.7–33.7)	15.0 (2.2–37.5)	0.588
CRP (mg/dL)	14.4 (5.4–25.9)	20.3 (9.6–29.7)	8.0 (3.5–18.4)	<0.001
Bacteremia	89 (53.9)	40 (51.9)	49 (55.7)	0.746
Vitamin C level (μmol/L)	12 (5.3–25.0)	5.1 (3.0–7.7)	23.4 (15.9–41.1)	<0.001
Thiamine level (nmol/L)	147.1 (99.6–217.5)	142.1 (98.0–237.7)	150.0 (106.3–208.1)	0.527
Thiamine deficiency *	12/141 (8.5%)	8/70 (11.4%)	4/71 (5.6%)	0.352
SOFA, initial	7 (6.0–10.0)	8.0 (7.0–10.0)	7.0 (5.5–10.0)	0.004
Adjunctive steroid use	94 (57.0)	44 (57.1)	50 (56.8)	0.999
Vitamin C and thiamine administration	91 (55.2)	45 (58.4)	46 (52.3)	0.523
MV	67 (40.6)	37 (48.1)	30 (34.1)	0.063
RRT	28 (17.0)	16 (20.8)	12 (13.6)	0.255
ICU admission	142 (86.1)	65 (84.4)	77 (87.5)	0.730

Data are shown as the median (IQR) for continuous variables or as a number (%) for categorical variables. * For thiamine, there are 24 missing values. IQR, interquartile range; WBC, white blood cell count; CRP, C-reactive protein; SOFA, Sequential Organ Failure Assessment; MV, mechanical ventilation; RRT, renal replacement therapy; ICU, intensive care unit.

**Table 2:** Comparison of primary and secondary outcomes between two groups.

Outcomes	Overall (*n* = 165)	Vitamin C Deficiency (*n* = 77)	Normal (*n* = 88)	*p*-Value
**Primary outcome**				
28-day mortality	30 (18.1)	18 (23.4)	12 (13.6)	0.083
**Secondary outcomes**				
7-day mortality	17 (10.3)	11 (14.3)	6 (6.8)	0.188
In-hospital mortality	35 (21.2)	20 (26.0)	15 (17.0)	0.096
Change in SOFA score (72-h)	3 (0–4)	3 (−1–5)	3 (0–4)	0.998
Shock reversal	139 (84.2)	61 (82.4)	78 (88.6)	0.368
Time to shock reversal (minutes)	54 (40–92.5)	54.5 (40–155)	53.5 (37.5–81.5)	0.510
Vasopressor-free day	12 (10–13)	11 (6–12)	11 (10–13)	0.199
MV-free day	14 (11–14)	14 (11–14)	14 (8–14)	0.057
ICU-free day	10 (6–12)	9 (4–12)	10 (5.5–11)	0.542
Hospital LOS (days)	13 (8–23.8)	13 (9–24)	13 (9–25)	0.932

Data are shown as the median (IQR) or as a number (%). IQR, interquartile range; SOFA, Sequential Organ Failure Assessment; MV, mechanical ventilation; ICU, intensive care unit; LOS, length of stay.

**Table 3:** Univariable and multivariable Cox regression analyses for 28-day mortality.

	Univariable		Multivariable	
Parameter	HR (95% CI)	*p*-Value	HR (95% CI)	*p*-Value
Vitamin C deficiency	1.80 (0.87–3.73)	0.115	2.65 (1.08–6.45)	0.032
IV administration of vitamin C and thiamine	1.73 (0.81–3.71)	0.155	1.58 (0.70–3.56)	0.271
Age, years	0.98 (0.96–1.01)	0.221	0.99 (0.96–1.02)	0.429
Chronic liver disease	2.04 (0.78–5.32)	0.146	1.01 (0.30–3.33)	0.991
Hematologic malignancy	1.19 (0.45–3.10)	0.728	0.55 (0.178–1.73)	0.310
Lactate (+1 mmol/L)	1.24 (1.19–1.38)	<0.001	1.25 (1.08–1.45)	0.003
CRP	1.00 (0.98–1.03)	0.763	0.98 (0.95–1.01)	0.211
Albumin	0.30 (0.16–0.54)	<0.001	0.33 (0.17–0.67)	0.002
SOFA, initial	1.27 (1.13–1.42)	<0.001	1.19 (1.05–1.34)	0.007

CI, confidence interval; HR, hazard ratio; IV, intravenous; CRP: C-reactive protein; SOFA, Sequential Organ Failure Assessment.

## Data Availability

The datasets used in this work are available upon reasonable request from the corresponding author and are not publicly available.

## Abbreviations

ROS	reactive oxygen species
RNS	reactive nitrogen species
ED	emergency department
SST	Serum-separating tube
BP	blood pressure
SOFA	sequential organ failure assessment
RRT	renal replacement therapy
MV	mechanical ventilator
ICU	intensive care unit
LOS	hospital length of stay
IQR	interquartile range
CRP	C-reactive protein
HR	hazard ratio
CI	confidence interval
SVCT-1	sodium-dependent vitamin C transporter-1
SVCT-2	sodium-dependent vitamin C transporter-2
